# How Does Economic Inequality Affect Infanticide Rates? An Analysis of 15 Years of Death Records and Representative Economic Data

**DOI:** 10.3390/ijerph16193679

**Published:** 2019-09-30

**Authors:** Seong-Uk Baek, Sung-Shil Lim, Jihyun Kim, Jin-Ha Yoon

**Affiliations:** 1 College of Medicine, Yonsei University, Seoul 03722, Korea; posososo@naver.com; 2 The Institute for Occupational Health, Yonsei University College of Medicine, Seoul 03722, Korea; LSSMAIL@yuhs.ac (S.-S.L.); JIHYUN0924@yuhs.ac (J.K.); 3 Department of Preventive Medicine, Yonsei University College of Medicine, Seoul 03722, Korea

**Keywords:** infanticide, inequality, economic recession, unemployment

## Abstract

Background: Is there a relationship between economic inequality and infanticide rates? Few studies have examined the socioeconomic factors that trigger infanticide. This study aims to statistically analyze the effect of these factors on infanticide rates. Methods: This study used infant death records in South Korea from 2003 to 2017 to assess the impact of unemployment rates and various statistical indicators (e.g., GDP and income inequality index) on the rate of infanticide. A generalized additive model and a quasi-Poisson regression were used for statistical analyses. Results: A time-trend analysis shows that the infanticide rate tended to grow despite a decreasing trend in the quarterly infant mortality rate. A 1% increase in the unemployment rate is associated with a significant rise in the relative risk of infanticide after a lag of two quarters. Relative risks increased significantly three and four quarters after a 0.1 rise in the p80/p20 ratio (income inequality index). Conclusions: Policymakers should pay attention to socioeconomic factors while formulating healthcare regulations to protect potential infanticide victims, including vulnerable infants and their parents.

## 1. Introduction

### 1.1. Question of Current Study

Is there a relationship between economic inequality and the rate of infanticide? According to the data released by Korean Statistical Information Service (KOSIS), as a single year, the risk of becoming a victim of murder is highest during the first year of life compared to all other ages. This phenomenon has been also reported in some Western countries [1]. Infanticide is defined as the intentional killing of infants, wherein the perpetrators are mostly parents or stepparents [2]. In South Korea, infanticide and the abandonment of infants have become important social issues. Infanticides have often been committed as a method of sex selection in South Korea, where the preference for sons is widespread [3,4]. The “baby box” in which infants could be abandoned safely, has triggered social debates [5]. While the infant mortality rate has gradually decreased, due to the improvement of perinatal medical care [6], only a few studies have investigated infanticide in South Korea. To date, no policy or legislative changes have significantly impacted infanticide rates.

### 1.2. Multiple Causes of Infanticide

Over the past decade, several studies have been conducted to better understand the causes of infanticide [7], mostly from psychiatric, sex selection, and socioeconomic perspectives. It has been found that infant killings, rather than being a senseless “crime of the devil”, are closely related to a variety of biological, psychological, and cultural factors [8]. Researchers have used animal studies to identify the potential biological causes of infanticide; for instance, genetic mutations have been associated with infanticide in pigs [9]. Studies that focused on psychiatric symptoms of human infanticide have found that perpetrators often suffer from psychological disorders [10,11], such as paranoid schizophrenia, postpartum psychosis, and personality disorders [12]. Sex selection (e.g., preferring male over female babies) is reported as one of the motivations for infanticide [13,14], highlighting that sociocultural factors also affect infanticide.

### 1.3. Environmental Causes of Infanticide

Recently, poor natural and social environments have been discussed as possible determinants of infanticide. For example, cold stress in mice has been shown to triple their tendency to commit infanticide compared to a control group [15]. This result might be related to survival instincts. A review article has identified exploitation, resource competition for food or nest sites, action to improve the parent’s survival chances or for sex selection to increase the chance of one’s own offspring’s survival as the causes of infanticide in the animal world [16]. This suggests that a poor natural survival environment increases the risk of infanticide.

Some research has claimed that the economic hardship experienced by parents is closely related to infanticide [17,18]. Married, poverty-stricken parents, who already have several children, have sometimes committed infanticide when they believed that they could not care for another child [11]. Gauthier et al. reported that areas with poor economic status have a high rate of parental infanticide [18]. Haapasalo et al. suggested that the economic burden experienced by parents is associated with infanticide [17]. Such investigations support the so-called “economic stress hypothesis” [18] which suggests that a heavy economic burden on parents is the main cause of infanticide. Therefore, economic factors, as well as parental psychiatric states, should be carefully reviewed in infanticide investigations.

### 1.4. Macro-Level Economic Variables and Their Associations with Infanticide

There have been studies that explored the relationship between macro-level socioeconomic variables and child homicide. Razali et al. suggested that economic and social inequalities are associated with higher infanticide rates. Gender inequality is positively correlated with infanticide rates, while the Human Development Index is inversely correlated with them [19]. Another article found that an increase in economic growth is associated with lower child homicide rates [20]. Yasumi et al. also suggested that unemployment rates had a significant relationship with filicide rates in Japan [21].

### 1.5. Hypothesis of the Current Study

Previous studies on the socioeconomic factors that affect infanticide did not elucidate the relationship between economic fluctuations and changing patterns of infanticide. The working hypothesis of this study is that macro-level economic variables have significant correlations with infanticide in a lag-time analysis. The present study is the first to statistically analyze the lag effect of changes in macro-level economic situations on the rate of infanticide. This study uses 15-year data on infanticide and various economic indicators, such as GDP, unemployment rate, and income inequality, to reveal hitherto unexplored causes of infanticide.

## 2. Materials and Methods

### 2.1. Data

This study acquired the quarterly death records of infants from the official death record certificates of all deaths in the South Korean population during the targeted period. In the case of infants, these records did not include the deaths of babies whose parents did not report their births to the government.

The Korean Classification of Disease (KCD), which coincides with the International Classification of Diseases, 10th Edition (ICD-10), was used to classify causes of deaths. Death caused by “assault” was defined by the ICD-10 codes X85–Y09. Stillbirths were excluded from infanticide since infanticide is coded as X85–Y09 in the ICD-10, while stillbirth is coded as P95. We focused on the rate of deaths by assault among infants under one year of age. The infanticide rate was defined as the number of quarterly infant deaths by assault per 100,000 infants born in each quarter.

All adopted socioeconomic variables, including quarterly GDP growth rate, unemployment rate, income inequality index (p80/p20 ratio), quarterly change rate of income in the bottom 20th percentile and 10th percentile of the population, and divorce rate, were obtained from the Korea National Statistical Office [22]. Quarterly GDP growth rate is defined as the percentage of change in real GDP from the previous quarter. The income inequality ratio, p80/p20, is defined as the ratio of income in the top 20% of the population to the income in the bottom 20%. The Gini coefficient is the most commonly used measure of the inequality of income distribution. However, since the KNSO announces the Gini coefficient yearly, not quarterly, another indicator of income inequality, p80/p20 ratio, was used in this study. Other studies have used this index to analyze the relationship between income inequality and public health [23,24].

The income inequality index, p80/p20 ratio, has been announced by KNSO since 2003, and the last death records of infants were announced in 2017. Hence, we set the study period from 2003 to 2017.

The data used in this study did not include any personal information. The Institutional Review Board (IRB) of the Yonsei University Health System approved the current study design (IRB number: Y-2017-0100).

### 2.2. Statistical Analysis

We used distributed lag non-linear models (DLNM) [25] for statistical analysis. DLNM is a useful statistical tool, which can be used to explore the delayed effects of exposure to environmental factors in a population [25,26]. In our model, the quarterly infanticide rate was regressed over socioeconomic variables while controlling for long-term trends and seasonal variation. The quarterly change of infanticide was adjusted for seasonal variation. A generalized additive model (GAM) was used for remnant trend fitting. The optimal degree of freedom of GAM spline parameter for minimal AIC was 4, and the AIC was 82.5. The autocorrelation plot of residuals showed no cyclic pattern. We used a function called “crossbasis” in R to examine the potential lagged effects of these variables. The main results were derived from the following equation:

Log (E [quarterly infanticide rate]) = cb (socioeconomic variables) + NS (long term trend, df = 4) + Quarter

where “cb” indicates the cross-basis function modeling polynomial distributed lag effect of socioeconomic variables, determined through the package “dlnm” in R for the DLNM. We analyzed the impact of economic variables over lags from zero to five quarters. “NS” denotes the nature spline function for adjusting the long-term trend. The variable named “Quarter”, numbered from 1 to 4, was adopted in our equation to adjust for the seasonal trend. Poisson-model fitting with deviance and degrees of freedom showed that there was an overdispersion problem; hence relative risk (RR) of infanticide and its 95% confidence interval (95% CI) were calculated using a quasi-Poisson regression model.

## 3. Results

**Figure 1:**
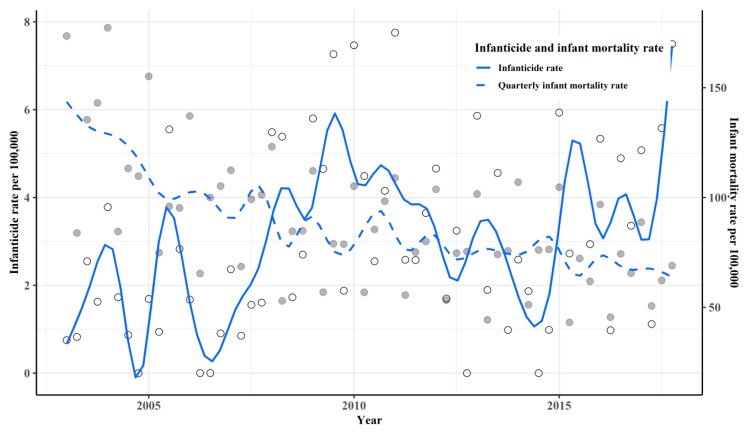
Time trend of infanticide and quarterly infant mortality rate per 100,000 from 2003 to 2017. The points colored in gray indicate infant mortality rate by quarter and the white point indicate the infanticide rate by quarter.

During the study period, there were 205 infanticides in total. The mean quarterly infanticide rate was 3.32 per 100,000. Figure 1 shows the rate of quarterly infant mortality and the time trend of infanticide from 2003 to 2017 in South Korea. During this period, the mean quarterly infant mortality rate was 88.59 (per 100,000).

**Figure 2:**
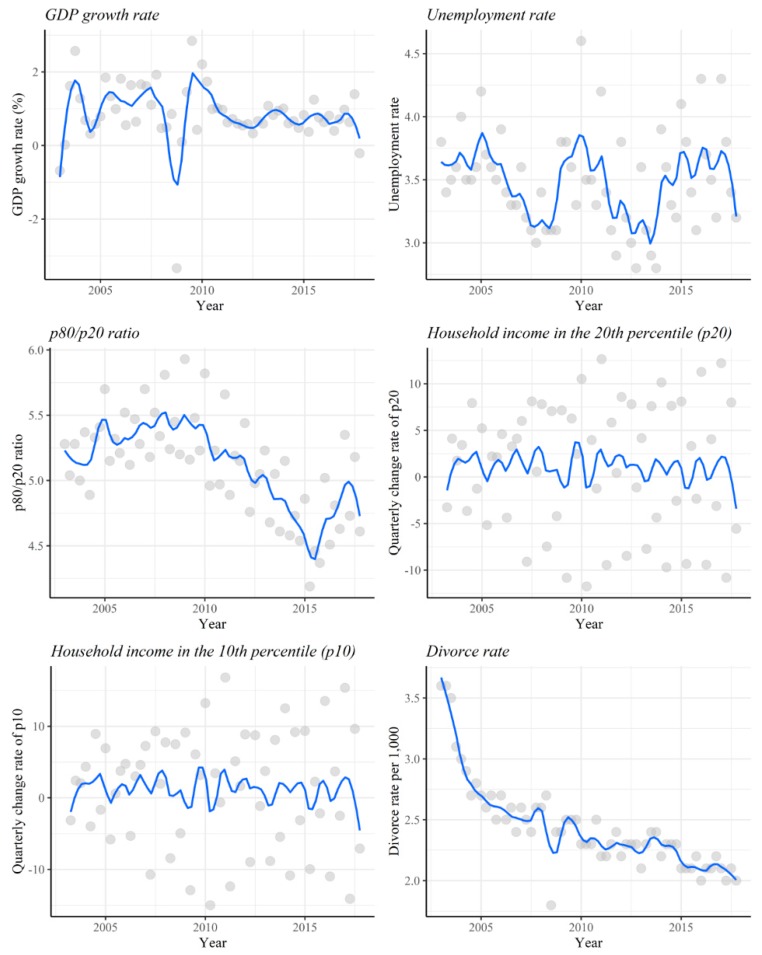
Time trend of socioeconomic variables from 2003 to 2017.

As Figure 1 shows, the time trend of the infanticide rate gradually increased, while the overall death rate of infants decreased. The infanticide rate peaked between 2009 and 2011, then fluctuated until 2014, before sharply increasing after 2015. In contrast, the quarterly infant mortality rate was highest in 2003 and decreased after that. Figure 2 shows the value and time trend of each socioeconomic variable during the study period by quarter. The peak value of the unemployment rate was 4.6 in the 1st quarter of 2010, and the p80/p20 ratio was 5.93 in the 1st quarter of 2009. Quarterly GDP growth rate hit its lowest point in the 3rd quarter of 2008. As Figure 1; Figure 2 show, from 2008 to 2010, when the economic recession took place in South Korea, there were overall increases in the unemployment rate, the income inequality index (p80/p20 ratio), and infanticide rates. Table 1 presents the definitions and descriptive statistics of the socioeconomic variables used in the current study.

**Figure 3:**
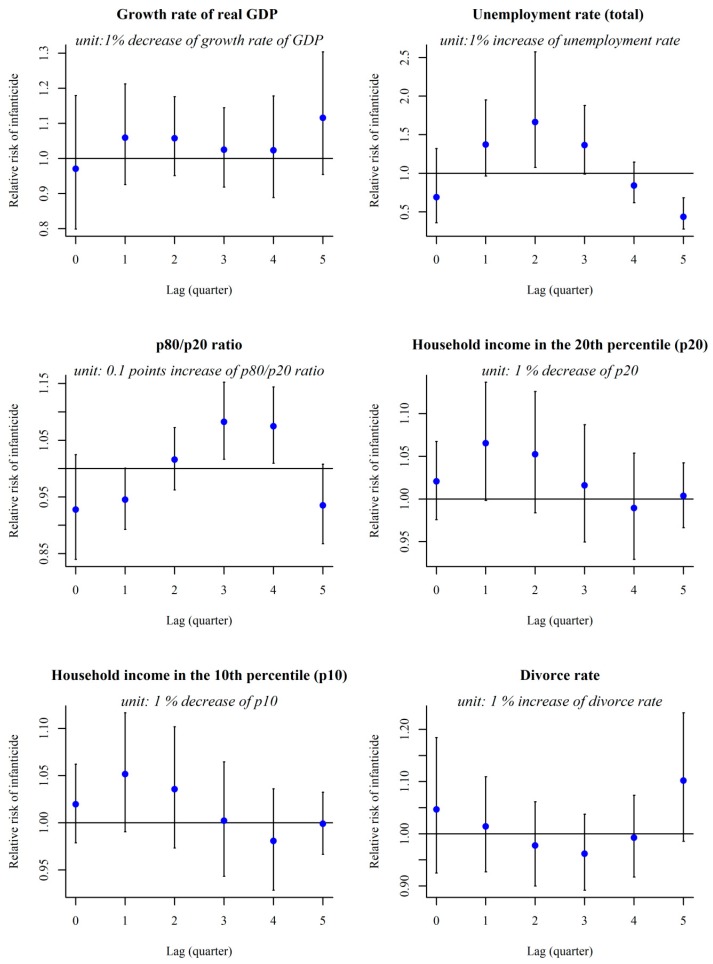
Time-lag effect of changes in socio-economic values on infanticide rate.

We conducted a lag-effect analysis of the effect of each socioeconomic variable on the infanticide rate (Figure 3). As Figure 3 shows, a decrease in the quarterly GDP growth rate was not related to an increase in the RR of the infanticide rate during the following five quarters. There was, however, an association between a 1% increase in unemployment rate and a significant rise in RR of infanticide after a lag of two quarters. The RR (95% CI) was 1.66 (1.08–2.57) at the 2-quarter lag. In lag 5, a harvesting effect was observed (RR: 0.44 [0.28–0.68]). There was also a time-lag relationship between the effect of income inequality and infanticide. RRs (95% CI) increased significantly three and four quarters after a 0.1 rise in the p80/p20 ratio (RR, 95% CI was 1.08, 1.01–1.14, and 1.07, 1.01–1.13, respectively). The were no significant relationships among infanticide and divorce rate, household income in the 20th percentile, and income in the 10th percentile.

## 4. Discussion

A time-trend analysis showed that the Korean infanticide rate has tended to grow despite the decreasing trend in the quarterly infant mortality rate. The present study has revealed a meaningful lag-time effect of economic indicators—unemployment rate and income inequality index—on infanticide since 2003 in South Korea. The time-lag analysis conducted in this study showed that a decrease in GDP growth rate was not related to infanticide, but that an increase in unemployment rate was significantly related to infanticide after two quarters. The increase in the income inequality index (p80/p20 ratio) is significantly associated with an increase in the infanticide rate after three and four quarters.

Infanticide occurs in both humans and animals. Cases of infanticide have been reported since ancient time in most societies [27]—in Japan, it is called mabiki (“thinning out of young shoots”) [28], in the High Arctic, infanticide is related to a preference for male hunters over females [13], and in China, it reflects Confucian culture’s preference for a male children [14]. Some scholars suggest that to understand infanticide, research should consider socioeconomic factors in addition to socio-cultural ones [1,7].

In the past few decades, various studies have been conducted to investigate the underlying causes of child abuse [29]. Economic hardship is well-known to be a risk factor for child maltreatment [30]. In addition, studies have identified a close relationship between macro-economic variables, such as the unemployment rate [31] or the Consumer Sentiment Index [32], and child maltreatment. The present study explored some socioeconomic determinants of infanticide and revealed that the unemployment rate and the income inequality index are important factors in the rate of infanticide.

The goal of this study was to understand how big-picture economic-environmental factors lead individuals to commit infanticide. The current investigation found that the unemployment rate has a significant time-lag effect on infanticide rates. Many studies have been done to investigate the possible effects of the unemployment rate on general public health [33]. Macro-level unemployment rates have been found to have a close relationship with suicide rates [34], cancer-mortality [35], and smoking behavior [36]. In addition, some researchers have explored the time-lag effect of the rate of unemployment on public health. Recently, a well-designed study found that an increase in the unemployment rate was significantly related to an increase in all-cancer mortality from zero to five quarters [35]. Another article reported that a decrease in the unemployment rate is associated with a short-term decrease in mortality in China [37]. The current study meaningfully explored the time-lag effect of an increase in the unemployment rate on infanticide rates. Some investigations have suggested that parental employment status is an important factor in the health of both parents [38] and infants [39,40]. Having a stable employment status is reported to lower the risk of postpartum depression [38]. Lindo suggested that parental unemployment status has negative effects on infant health [39]. The findings of the present study suggest that there is an association between the unemployment rate and the infanticide rate, which is in keeping with previous scholarship.

Second, this study found that income inequality has a significant time-lag relationship with infanticide rates. Inequality has become an important indicator of public health [41]. Several studies have found that the Gini coefficient is more influential than absolute wealth indicators, such as GDP, in affecting public health issues. One article suggested that the Inequality-Adjusted Human Development Index better predicts the infant mortality rate worldwide than the Human Development Index [42]. Income inequality, represented by the Gini coefficient, also has a close relationship with public mental health [43]. Alongside these works, the results of the current study elucidate that both social inequality and the unemployment rate are associated with changes in the infanticide rate, but household income in the 20th and 10th percentile and divorce rate did not have any significant relationship with infanticide. Several previous studies conducted analyses of the lag effect of the income inequality index on public health [44,45], reporting that an increase in income inequality is related to adverse health outcomes in a lag-effect model [45]. However, little research has been conducted to examine the impact of inequality on infant health. The present study elucidates the pattern by which inequality affects infanticide over time.

Like employment status, income inequality is a significant factor that affects the health of both parents [23,46,47] and infants [48,49,50]. In terms of reproductive health, several studies reported that parents with low socioeconomic status are less likely to receive adequate medical support during and after pregnancy [51,52]; furthermore, they have a higher risk of both postnatal depression [53] and postpartum psychosis [54], which are considered important causes of infanticide. Furthermore, a systemic review found that health outcomes of infants with deprived parents are significantly worse [50] that those with economic stability. The current study also found that there is a significant increase in the rate of infanticide when income inequality increases, following a specific lag time.

Generally, the GDP growth rate was inversely related to unemployment rates (Okun’s law) [55]. In this study, the unemployment rate was related to infanticide fluctuation, but GDP was not. There were reports of a mismatch between the GDP growth and unemployment rates [56]. A study of South Korea after the global financial crisis in 2008 observed less significant relations between GDP growth rate and unemployment [57]. In addition, while some researchers found that economic growth was negatively related to income inequality [58], such a relationship is quite controversial [59]. Further in-depth studies are needed to elucidate the correlations between these economic variables and their effects on child homicide.

Both infant mortality and infanticide are affected by the variable socioeconomic status of a population [60,61]. The current study shows that the infant mortality rate decreased over the study period, while infanticide did not follow the same pattern. The decreased infant mortality rate over the study period is attributed to the gross improvement of perinatal and maternal health care services [6]. However, the infanticide rate could be affected by more complicated psychosocial relationships between individuals and society [51]. Income inequality is an important factor in that interaction, which in turn affects the behavior patterns of individuals [62]. For example, macro-level income inequality is positively correlated with child maltreatment in the US [63]. This study suggests a possible explanation for the fluctuation of infanticide rates despite the overall decrease in the rate of infant mortality.

This study has several limitations. First, there is an underestimation problem. If parents do not report the birth of their child, the child’s death cannot be properly reflected in statistics. These “silent deaths” have cause social consternation and debate [64] when they are revealed, for instance, through police investigation, but no official statistics are maintained on them. In addition, some babies who are killed directly after born alive could be classified as ‘stillbirth’, which is not included in our study. The misclassification error may have been reduced by the study’s use of ICD-10 codes to define infanticide, but no information is available on the number of unreported infant deaths. Despite this problem, this study still proves that economic factors affect infanticide rates. Although the literature on infanticide suggests that the perpetrators are mostly parents or stepparents, this study did not collect any direct information about perpetrators. Various studies suggest that psychological problems are one of the most important causes of infanticide, but, due to a lack of information, this study did not control for them. Finally, only 205 infanticides occurred during the study period. Such a small sample size can aggravate the stability of statistical results, and also makes it difficult to conduct further analyses that consider gender or geographical stratification. More comprehensive research is needed to investigate the relationship between macro- and micro-level factors that affect the infanticide rate.

In conclusion, our study has highlighted the relationship between infanticide and economic indicators, such as the unemployment rate and the income inequality index. Based on this finding, the current study proposes an answer to the question, “How does economic inequality affect infanticide rates?” Income inequality plays an important role in children’s public health [65]. For example, child mortality rates are positively correlated with a country’s macro-level income inequalities [62]. Furthermore, income redistribution is reported to positively affect children’s health outcomes in various ways [66,67]. This study suggests that policies to lower infanticide rates might work best when they consider the respective society’s macro-level income distribution.

The results of this study revealed that unemployment rates and income inequality have a meaningful lag-time effect on infanticide. Policymakers should take this into account to protect vulnerable infants and their parents from falling prey to infanticide since both groups are victims of desperate economic situations.

## Figures and Tables

**Table 1:** Definitions and characteristics of socioeconomic variables.

Variable	Definition	Median (Q1–Q3)	Min–Max
Infanticide rate per 100,000	number of infant deaths caused by assault per 100,000 infants	2.70 (1.68–4.98)	0.75–7.75
Quarterly growth rate of GDP (%)	percentage change of real GDP from previous quarter	0.77 (0.58–1.25)	−3.33–2.84
Unemployment rate (%)	number of unemployed people divided by the number of economically active persons aged 15 or older	3.5 (3.2–3.7)	2.8–4.6
Income inequality index			
p80/p20 ratio	the ratio of income in the top 20% of the population to income in the bottom 20%	5.17 (4.88–5.34)	4.19–5.93
Quarterly change rate of household income			
in 20th percentile	quarterly change rate of income in the bottom of 20% of the population	2.45 (−4.29–7.10)	−11.75–12.65
in 10th percentile	quarterly change rate of income in the bottom 10% of the population	2.39 (−5.14–7.62)	−14.98–16.81
Divorce rate (%)	number of divorces per 1000 people	2.4 (2.2–2.6)	1.8–3.6
